# Developing and evaluating a culturally-appropriate food kit for increased access to fruits and vegetables and DASH eating plan alignment in immigrant Hispanic/Latine individuals with hypertension: a pilot study

**DOI:** 10.1186/s40795-025-01089-z

**Published:** 2025-05-16

**Authors:** Ambria Crusan, Kerrie L. Roozen, Clara Godoy-Henderson, Angela Evans, Katie Reeves

**Affiliations:** 1 Department of Nutrition and Dietetics, Henrietta Schmoll School of Health Sciences, St. Catherine University, 2004 Randolph Avenue, St. Paul, MN USA; 2 Department of Health Services Research, Policy and Administration, School of Public Health, University of Minnesota, Minneapolis, MN 55455 USA

**Keywords:** Medically tailored groceries, Hispanic nutrition, Nutrition intervention, Hypertension, DASH diet, Cardiovascular disease

## Abstract

**Background:**

Effective nutrition interventions for hypertension (HTN), including the Dietary Approaches to Stop Hypertension (DASH) Eating Plan, fail to consider cultural preferences and barriers to obtaining and utilizing fruits and vegetables (F/V). A paucity exists in the literature regarding nutrition interventions tailored for Hispanic/*Latine* communities. This project aims to determine the outcomes associated with improved access to a culturally-appropriate, medically-tailored foods for Hispanic/*Latine* individuals with HTN via an iterative process: 1) conceptualization of culturally-appropriate F/V for a DASH box using a patient/provider survey, 2) formative DASH box development utilizing individual interviews for food preference feedback, and 3) free-living pilot trial of DASH box intervention to determine impacts on cardiometabolic markers.

**Methods:**

Using community-based participatory research methods, findings from 50 surveys revealed F/V preferences which supported the conceptualization of 6 boxes, including F/V and staple foods to encourage DASH Eating Plan adherence. Boxes were displayed during 15 interviews gathering feedback on acceptability. Themes were assessed using the Framework Method and finalized via consensus building. A 28-day open trial enrolling 21 participants collected pre- and post- measurements of blood pressure (BP), weight, waist circumference (WC), and skin carotenoid levels. Weekly DASH boxes and diet education were provided. Pre-to post-changes in cardiometabolic markers were calculated via t-tests.

**Results:**

Thematic analysis determined participants prefer fresh F/V, use staple items to compliment F/V, and experience barriers (time, money, transportation) to accessing or using F/V. Post intervention, there was a significant improvement in systolic BP (mean difference of -4.1土7.8 mmHg, p = 0.01), diastolic BP (-3.7土6.4 mmHg, *p* = 0.004), and WC (-0.8土1.1 inches, *p* = 0.003). While mean difference in weight (-1.2土4.8 pounds, *p* = 0.26) and skin carotenoid levels (26.7土74.1, *p* = 0.06) changed, results were not significant.

**Conclusions:**

This pilot study provides formative contributions regarding culturally-appropriate interventions for chronic disease management, suggesting a medically-tailored DASH box may be effective in lowering BP and other cardiometabolic risk factors for Hispanic/*Latine* individuals with HTN.

**Clinical trial registration:**

ClinicalTrials.gov, Identifier NCT05802134, Registered 3/24/2023, https://clinicaltrials.gov/study/NCT05802134.

**Supplementary Information:**

The online version contains supplementary material available at 10.1186/s40795-025-01089-z.

## Introduction

Cardiovascular disease (CVD) entails conditions affecting the cardiovascular system, with hypertension (HTN) distinguished by systolic blood pressure (BP) ≥ 140 mmHg and/or diastolic BP ≥ 90 mmHg [1]. CVD is a leading cause of death in Hispanic Americans [1, 2], with HTN affecting approximately 44% of Hispanic/*Latine* individuals in the United States (US)[3]. Relevant CVD risk factors for the Hispanic/*Latine* population include under-representation in HTN literature across Hispanic ethnicities, smoking, insufficient physical activity, dietary patterns, increased body weight, and suboptimal cardiometabolic markers [2, 4]. Although Hispanic/*Latine* individuals have a lower HTN prevalence than non-Hispanic white individuals, they are less likely to be diagnosed or seek medical treatment [1]. Moreover, uncontrolled HTN and poor intervention compliance is highest in US Hispanic populations [1, 5].

Dietary strategies for the prevention and management of HTN are supported by a robust body of research [6, 7]. The Dietary Approaches to Stop Hypertension (DASH) Eating Plan is one dietary intervention that significantly reduces BP and other CVD risk factors such as cholesterol and body weight [7, 8]. The DASH Eating Plan recommends individuals consume 4–5 daily servings of both fruits and vegetables (F/V) and emphasizes utilizing whole grains, vegetable oils, legumes, fish, and poultry in substitution for refined grains, animal fats, and red/processed meats [9]. However, present inequities rooted in structural racism such as employment, education, targeted marketing of unhealthy foods, limited federal resources, neighborhood segregation, acculturation, and food apartheid, exacerbate disproportionate burdens of food insecurity and health literacy for the US immigrant Hispanic/*Latine* population, challenging adherence to DASH Eating Plan recommendations [6, 10–14].

Additional barriers to obtaining foods to promote adherence to the DASH Eating Plan include lack of culturally-appropriate food access and availability, difficult interactions related to language barriers, time constraints, discrimination, and documentation status [6, 15]. Food-at-home prices in the US have increased by 18.4% between 2022 and 2024 [16]; the difficulties in food affordability are heightened for individuals with low incomes. Culturally-appropriate nutrition interventions targeting immigrant Hispanic/*Latine* patients are sparse, despite the apparent need [17, 18]. Therefore, nutrition interventions designed to address personal and structural barriers to F/V consumption faced by the Hispanic/*Latine* population may benefit cardiovascular health on an individual, familial, and community health level [14, 19].

Medically-tailored meal kits designed to increase compliance and reduce barriers to food access are a recent advancement in nutrition interventions [4, 20–24]. The body of literature surrounding medically-tailored meals and/or groceries is expanding, with evidence supporting improved health metrics as a result of participation in medically-tailored interventions [4, 23, 25]. Additionally, Hager et al. evaluated 9 US prescription produce programs, with findings supporting increased F/V consumption, decreased systolic and diastolic BP, lowered hemoglobin A1 C values, and reduced odds of experiencing food insecurity [4]. Well-designed studies are still necessary to support and validate the effectiveness of these interventions [18]; a gap in the literature exists regarding the acceptability and validity of medically-tailored meal kits in Hispanic/*Latine* populations.

This project contributes to the small body of research on effective nutrition interventions for cardiovascular health in Hispanic/*Latine* populations. The objective of this study is to utilize community-based participatory research (CBPR) methods to determine the effect improved access to culturally-appropriate F/V to meet the DASH diet recommendations has on immigrant Hispanic/*Latine* individuals with HTN via a phased, iterative process: Phase 1) initial conceptualization of F/V to be included in a culturally-appropriate, medically-tailored"DASH box"using a patient/provider survey, Phase 2) formative intervention development utilizing individual interviews to gather qualitative feedback on the DASH box food contents, and Phase 3) pilot testing the weekly distribution of a DASH box to determine the effect on cardiometabolic markers, specifically systolic and diastolic BP and markers of adiposity, in a free-living clinical trial.

### Phase 1: initial conceptualization—patient/provider survey

#### Phase 1 methods

##### Survey design, participants, and recruitment

Phase 1 utilized a literature-informed survey to conceptualize the F/V contents most relevant for a culturally-appropriate nutrition intervention meeting the DASH Eating Plan parameters. This survey (supplement 1) encompassed validated questions regarding demographics [26, 27], food security status [28], and medical history [26]. The research team developed additional qualitative questions, due to inadequate validated tools on this subject, to gain insights on general food preferences/allergies, frequently consumed F/V, and hierarchical ranking of top-five preferred F/V to eat by themselves or to mix in with other dishes from each participant. All survey questions were professionally translated from English to Spanish and back-translated for clarity by certified interpreters.

##### Data collection

Between June and October of 2022, 50 participants from a community clinic were recruited during clinic hours to complete the 22-question survey in their preferred language (Spanish or English). Interpreters were onsite during administration to support any clarifications. Surveys were collected via paper and added to the Research Electronic Data Capture (REDCap) [29, 30] electronic tool hosted at St. Catherine University for data management. Inclusion criteria for participation required active involvement in the partner community medical clinic (*i.e.* patients, providers, medical interpreters), being ≥ 18 years, and identifying as Hispanic/*Latine*. Participants who declined participation or were not part of the community health clinic were excluded. Each participant received and agreed to informed consent to participate prior to engagement, in which participants were informed their ability to receive healthcare would not be compromised based on their participation. The principal investigator provides care at one of the community clinic sites, therefore, exclusion criteria included participants with relationships to the principal investigator pre-study study. The entirety of the following research was approved for human ethics by St. Catherine University’s Institutional Review Board #1744.

##### Data analysis

The demographic data analysis for this phase utilized REDCap [29, 30] and RStudio (Version 2022.07.2) RStudio Team [2022] [31] was used to assess correlation via Pearson’s correlation. Food security outcomes were determined via the US Department of Agriculture’s (USDA) Six-Item Short Form of the Food Security Survey [28]. Results were determined by binary outcomes of food security, using a yes/no model [32] for responses to the first two questions; tiered food security analysis of “high or marginal security”, “low security” or “very low security” to determine nuances in food insecurity status [28, 32]. All responses were coded and analyzed according to the measures provided by the survey [28].

#### Phase 1 results

Of the 50 participants who completed the survey, the average age of the 38 women (76%) and 12 men (24%) completing the surveys was 44.6 ± 14.4 years, and Mexico was the primary country of origin (*n* = 36). Other countries of origin represented were El Salvador, Ecuador, Guatemala, Cuba, Venezuela, and Puerto Rico. Many participants surveyed had an education level of high school graduates or below (48%). Of note, 28% of the sample refused to report their educational attainment.

In the patient sample, 47.7% reported experiencing food insecurity within the last 12 months via the binary measure of food security, and 61.4% indicated they were unable to afford healthful foods for balanced meals. Six participants (12%) were omitted from the food insecurity analysis with incomplete screeners or refusal to answer. In the tiered food security measure, the extent of food insecurity is clearer. High or marginal food security was present for 53.3% of the sample, whereas 44.4% of respondents reported experiencing low food security and 2.2% experiencing very low food security. This sample showed no significant correlation between education level and binary food security outcomes.

**Fig. 1:**
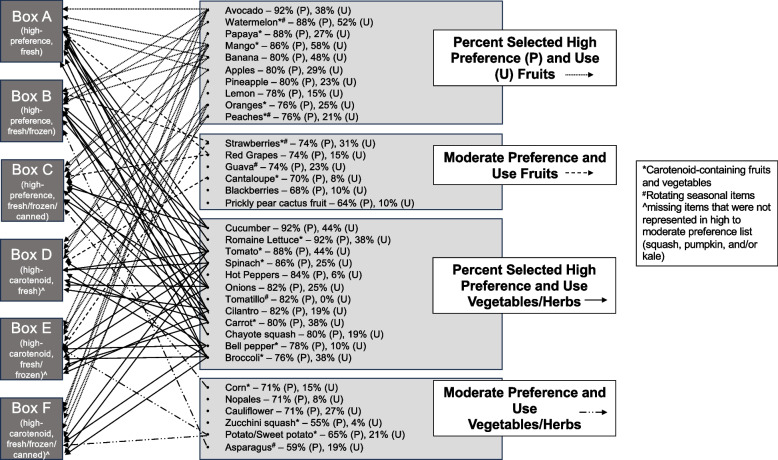
Fruits and vegetables by preference and use and their distribution amongst the 6 DASH boxes

Fruits with the highest preference, determined by reporting to be consumed if they were available, were avocado (92%), watermelon/papaya (88%), and mango (86%), while the most preferred vegetables were cucumber/lettuce (92%), tomato (88%), and spinach (86%). These results labeled “high-preference” F/V were used in the development of the DASH boxes. The fruits ranked as most preferred to eat by themselves or to mix in with other dishes, which we refer to as “high-use” items were mango (58%), watermelon (52%), and banana (48%), and the highest reported vegetables consumed included tomato/cucumber (44%) and lettuce (38%), paralleling some of the preferences. F/V showing lower preference were ground cherries (24%), starfruit (26%), collard greens/kale (31%), and peas (24%). Figure 1 displays additional high-preference and high-use F/V.

### Phase 2 formative evaluation—patient qualitative interviews

#### Phase 2 methods

##### Formative DASH box development

This formative phase consisted of developing a foundational medical nutrition therapy DASH box to satisfy cultural preferences aligning with DASH Eating Plan parameters. Feedback was received regarding box contents prior to administration in the open clinical trial (Phase 3). F/V preference results from Phase 1 community surveys informed DASH boxes used for demonstration during qualitative interviews in Phase 2. Researchers (ACC and KLR) developed six F/V boxes of varying modalities (fresh, frozen, and/or canned/packaged) of high- to moderate-preference F/V to support compliance to the required 8–10 servings of F/V for the DASH Eating Plan (Fig. 1). Three of six boxes had increased carotenoid content to support higher antioxidant activity.

##### Participants and data collection

Fifteen participants recruited from convenience sampling from Phase 1 or by Community Health Worker referral participated in one-hour, ethnographic interviews between October 2022 and January 2023 to explore individual perceptions of the appropriateness of the 6 DASH boxes (labeled A-F). Contents of the boxes were assessed via qualitative questions, allowing participants to scrutinize or validate each item. Participants were asked to rank boxes in order of preference from highest to lowest. Preceding each interview, participants completed a demographic and medical history [26, 27], food insecurity [28], and F/V intake survey [33, 34]. The inclusion and exclusion criteria mirrored Phase 1, with additional inclusion criteria of patients currently managing a chronic disease(s) such as diabetes, cardiovascular diseases, or obesity. Additional exclusion criteria were that participants must be a patient, not staff at the partner community health clinic. All participants provided informed consent to participate, and provided oral or written consent prior to the interview starting [35].

##### Data analysis

The interviews were assessed via Framework Matrix Analysis as previously described in our published work [35]. All interviews were recorded, transcribed verbatim, and translated to English (if necessary). Five interdisciplinary research team members familiarized themselves with the interviews, developed thematic codes, and met to achieve consensus required for thematic map development. Thematic framework was applied to all interviews, adding additional themes or sub-themes as necessary. The data was charted to the Framework matrix, and outcomes were interpreted [35]. The demographic data analysis for this phase utilized REDCap [25, 26] and RStudio (Version 2023.03.0.) RStudio Team [2023] [36].

#### Phase 2 results

##### Participant demographics

For participants completing the interview (*n* = 15), the average age of the 12 women and 3 men completing the surveys was 54.8 ± 8.8 years, and Mexico was the primary country of origin (*n* = 11). Other countries of origin represented were Ecuador (*n* = 1), Guatemala (*n* = 1), Venezuela (*n* = 1), and Puerto Rico (*n* = 1). In the sample (*n* = 14), 50.0% reported experiencing food insecurity within the last 12 months via the binary measure of food security and 78.5% indicated they were unable to afford healthful foods for balanced meals. One participant refused to report food security status [35].

##### Emergent themes

Thematic analysis related to the formative DASH boxes shown in the interviews determined participants prefer fresh F/V, in comparison to frozen or canned items, in tandem with staple items to complete a meal. Participants expressed personal experiences with barriers to F/V consumption and access, which included time, money, and transportation [35].

##### Reaction to the DASH box feedback

Participant input regarding ranked preference and cultural appropriateness was aggregated, with Box A being considered culturally-appropriate by 100% of participants and the primary preference of 67%. Box A was composed of entirely fresh and predominantly high-preference, high-use F/V. Additional staple items garlic, onions, rice, beans, *tortillas*, cilantro, and *masa* flour were requested by participants to promote participation in DASH diet recommendations of whole grains, legumes, and daily consumption of 8–10 servings of F/V [35]. Participants also expressed a desire for weekly item rotation, leading our research team to develop a F/V needs survey, allowing participants to choose from a variety of available items, to be completed weekly during Phase 3.

### Phase 3: pilot testing—free-living, open clinical trial

#### Phase 3 methods

##### Overview

Participants with a body mass index (BMI) categorized as overweight (BMI 25–29.9) or obese (BMI > 30 kg/m2) and diagnosed with HTN were recruited for the DASH box intervention clinical trial. Participants received supplemental, culturally-appropriate F/V over a 28-day period [37]. Boxes provided on Day 0, Day 7, Day 14 and Day 21 contained a week’s worth of F/V to achieve 8–10 servings per day for participants, supported by education for chronic disease management. Encouragement and support to consume the F/V via recipes and weekly compliance text/call check-ins were conducted (Day 7, 14, 21). Assessment of cardiometabolic markers, demographics, and perception of food insecurity were collected on Day 0 and Day 28 of the intervention. This study was registered 3/24/2023 with ClinicalTrials.gov (NCT05802134, https://clinicaltrials.gov/study/NCT05802134).

##### Intervention DASH box development

The intervention DASH boxes were designed to align with recommendations for the DASH Eating Plan and provide 8–10 servings of F/V daily for the participant, as well as supportive F/V for additional household members. Any drug-nutrient interactions were pre-determined for each participant based on their medication reporting. In the weekly development of the DASH boxes, the F/V needs survey was constructed to accommodate patient's preferences for specific items (e.g. corn versus flour *tortillas*) and reflect availability at a local food shelf (potatoes, onions, apples, pears, rice, beans, corn tortillas, corn flour), Hispanic market (*chayote*, corn, *tortillas* and *nopales*), and grocery stores (all other F/V). Table 1 describes decisions to include certain items in the intervention DASH box.

**Table 1:** DASH box development decision sourcing for a culturally-appropriate, medically-tailored nutrition intervention

	**Decision Source**		
**Intervention Component for Food Box Sourcing**	**Phase 1: Patient/Provider Survey**	**Phase 2: Patient Qualitative Interviews**	**Registered Dietitian Expertise **
DASH Eating Plan item alignment			X
High- and moderate-preference F/V	X		
Inclusion of staple food items to complete a meal		X	
Carotenoid-containing F/V for heart health			X
Weekly check-ins for compliance and participant support		X	X
F/V needs survey to include participant preferences each week		X	

Food sourcing and DASH box packing was conducted each week 1–2 days prior to the box delivery; all food and prepared boxes were stored to ensure food safety at the university’s food lab. Foods were sourced from multiple grocery stores (discount grocery stores, chain grocery stores, and Hispanic grocery stores) and an on-campus food shelf with access to quality, low-cost produce. Each box was budgeted to approximately $15.00 US dollars per week to ensure this would be a sustainable intervention for participants outside of the study. Participants'F/V needs survey was reviewed to ensure item alignment during sourcing and packaging. Each week, the total ounces of each F/V and staple item were determined using a standard electric kitchen scale. Servings provided to participants were calculated based on predetermined servings of each F/V item directed by total ounces. The total carotenoid content of the F/V was also calculated based on predetermined values per weight of each item.

##### Participants and demographic data collection

**Fig. 2:**
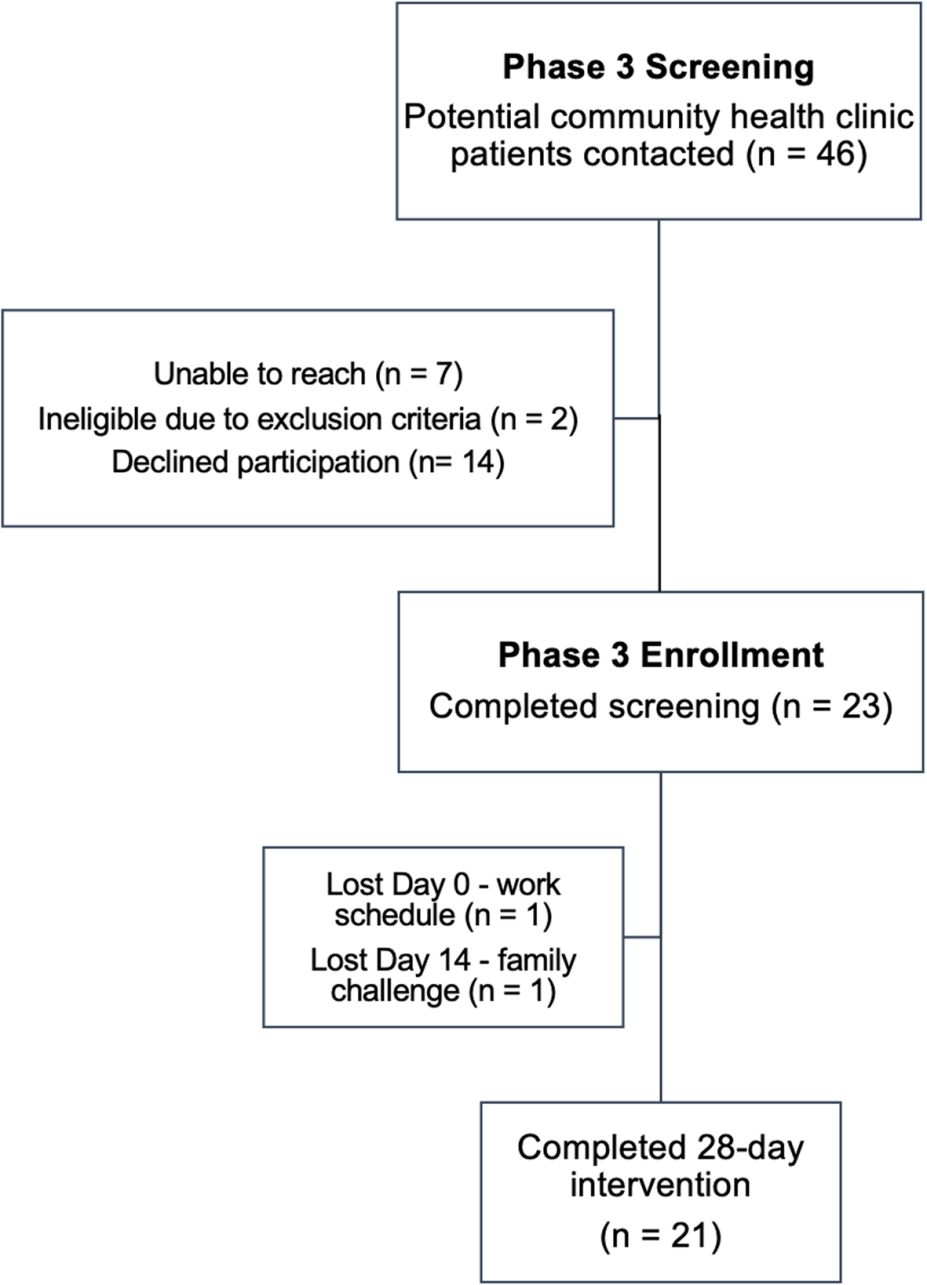
Flowchart of participants enrolled in Phase 3

Participants were recruited via flier, participation in previous phases of the study, or by Community Health Worker referral to participate in the 28-day open clinical trial to evaluate the effects of a DASH box. A demographic and medical history [26, 27], food insecurity [28], and F/V intake survey [38, 39] was completed by the final 21 study participants (Fig. 2). All participants were pre-screened over the phone prior to in-person screening to ensure eligibility. The inclusion and exclusion criteria mirrored those of Phase 2, with additional criteria of a BMI < 25, managing hypertension (must currently be managing HTN with medication or have either systolic BP > 140 or diastolic BP > 90), a non-smoker, and not using injectable insulin to manage diabetes mellitus if this comorbidity is present. Participants were excluded if they had any changes in medication to manage their BP in the past 3 months. Each participant provided written informed consent to participate prior to the intervention screening. All materials were available in Spanish and English, per participant preference. An interpreter was available for all medical appointments, as 95% of study participants were non-English speaking. All participant appointments were held at the most convenient health clinic site for participants.

##### Measures

On Day 0 and Day 28, cardiometabolic markers were collected in a private space using clean equipment. Cardiometabolic markers measured included systolic and diastolic BP, height, weight, waist circumference (WC), and skin carotenoid status. BP measurements were taken using the Omron BP monitor model BP785 while sitting after a 5-min rest period. Two BP readings were measured and averaged [8]. Height was collected with the participant’s back to the height rule. The back of the head, shoulder blades, buttocks and heels were in line with the stadiometer, with weight evenly on both feet and arms hanging loosely by their side. Body weight was measured using a standing digital scale, on a flat surface, with shoes and jacket or sweatshirt removed. The participant’s BMI was calculated based on their height and weight. WC was measured via locating and marking bony landmarks above the iliac crest; the horizontal WC measurement was measured and recorded in inches [40]. Skin carotenoid status was assessed as an indicator of F/V intake [41, 42] via an average of three non-invasive skin spectrometry readings using a VeggieMeter (Longevity Link Corporation, UT). A 10-s measurement of the participant’s fingertip involved contact with a lens, and a temporary blood clearing of measured tissue via a small compression arm. The VeggieMeter corrects for oxy-hemoglobin and melanin, providing scores that parallel tissue carotenoid concentrations [43].

##### Intervention summary

In-person screening was conducted prior to Day 0 to ensure participants met eligibility criteria. Participant preferences for their Day 0 DASH box were gathered. Participants were provided a study overview, completed a consent form, demographic and medical history form, and a F/V intake screener. Anthropometric measures of height, weight, and BP were collected. Participants were asked to complete a F/V needs survey at the screening or five days before Day 0 if not completed upon screening. The F/V needs survey determined the number of members in the household being supported by the DASH box and their fruit, vegetable, and staple preferences for the following week. Participants could decline items and indicate which predetermined alternative they preferred. Staple items to support the use of F/V and increase compliance to DASH recommendations included rice, beans (dried or canned), onions, garlic, cilantro, *tortillas*, and *masa*. Participants indicated preferences for white or brown rice, dry or canned beans, and black, pinto, or red beans.

On Day 0 of the intervention, participants returned to the clinic for collection of cardiometabolic markers and nutrition education. The educational materials were developed with an interprofessional approach to HTN management co-developed by Occupational Therapists and Registered Dietitians with the intention to yield increased compliance to dietary changes and currently prescribed medications. Educational packets were provided with verbal education surrounding HTN parameters, serving sizes and eating patterns aligning with the DASH Eating Plan (for use in patient serving size reporting), and sodium guidelines. Management of stress, medication adherence, weight, and physical activity participation were emphasized as tools to reduce BP. The education was conducted in the participant’s preferred language (Spanish or English) by two to three members of the research team, two of which have expertise in the field of nutrition and dietetics (ACC- Registered Dietitian and KLR- Dietetic Intern), and three who served as interpreters (AR, MP and CGH) when needed.

Days 7, 14, and 21 compliance check appointments were conducted by the research team (ACC, AFC, AR, KLR, CGH) via text or phone call, per patient preference. Participants completed a F/V compliance survey and a F/V needs survey for the following week. The F/V needs survey was conducted to re-gauge fruit, vegetable, and staple preferences with consideration of varying factors in availability and seasonality. Support was provided at assessment appointments in the form of recipes, as needed. All participant questions were fielded by a designated member of the research team, and data collection regarding patient ability to consume 8–10 servings of F/V daily was conducted. DASH boxes were provided to participants weekly via home delivery or clinic site pick-up per individual preference. At the last appointment (Day 28), a F/V compliance survey and repeat measures for cardiometabolic markers were collected. All components of the intervention were delivered as planned.

##### Data analysis

This pilot intervention assessed feasibility and effectiveness of the DASH box; therefore, analysis focused on descriptive characteristics of the sample and change in cardiometabolic markers pre- and post-intervention. REDCap [29, 30] was utilized to analyze sample characteristics via means (standard deviations) and percentages. RStudio (Version 2023.12.1) RStudio Team [2023] [36] was used to calculate t-tests (significance p < 0.05) and effect sizes to determine the clinical magnitude of our preliminary outcomes.

#### Phase 3 results

##### Participant demographics

For participants completing the open clinical trial (*n* = 21), the average age was 49.8 ± 9.2 years, and races were reported as black (*n* = 1), white (*n* = 9), Native American Aztec (*n* = 4), and more than 1 race (*n* = 7). All participants reported being of Hispanic/*Latine* ethnicity with Mexico being the primary country of origin (*n* = 15). Other countries of origin represented were Ecuador (*n* = 2), El Salvador (*n* = 1), Honduras (*n* = 1), Venezuela (*n* = 1), and Puerto Rico (*n* = 1). The average number of years in the US was 18.1 ± 9.1, ranging from 2 months to 31 years. On Day 0 (*n* = 17), 64.7% reported experiencing food insecurity within the last 12 months via the binary measure of food security and 81.3% indicated they were unable to afford healthful foods for balanced meals. Four participants refused to report their food security status. On Day 0, 19% of participants reported enough F/V in their home to allow consumption of 8–10 servings daily. Additional participant demographics, average F/V intakes prior to the intervention, Global Physical Health T-Score and Global Mental Health T-Scores are reported in Table 2.

**Table 2:** Participant characteristics for phase 3: pilot testing - free-living, open clinical trial

**Demographic Characteristics**	**N (%)**	**Average number of servings fruits/vegetables per day (SD)**^a^	**Average number of servings of fruits/vegetables per day, without dried beans (SD)**	**Physical Health T-score (SE)**^b^	**Mental Health T-score (SE)**^b^
**(n=21)**		2.3 (1.8)	1.9 (1.4)	41.3 (4.2)	45.3 (3.6)
Biological Sex					
*Men *	12 (57.1)	1.9 (0.7)	1.7 (0.8)	43.6 (4.3)	46.2 (3.6)
*Women *	9 (42.9)	2.6 (2.3)	2.0 (1.8)	39.7 (4.2)	44.8 (3.7)
Employment Status					
*Employed*	16 (76.2)	2.1 (1.6)	1.6 (1.2)	40.7 (4.2)	43.3 (3.6)
*Unemployed/Retired/Temporary Employment *	5 (23.8)	3.0 (2.5)	2.5 (2.1)	43.4 (4.4)	48.9 (3.7)
Food Security Status ^c^					
*Low Food Security *	10 (55.6)	3.0 (2.3)	2.2 (1.8)	40.5 (4.2)	43.5 (3.7)
*High/Marginal Food Security *	8 (44.4)	1.9 (1.2)	1.8 (1.2)	43.9 (4.3)	47.1 (3.6)

^a^Eating At America’s Table Study Quick Food Scan [32] assessed via the National Cancer Institute’s Scoring Guidelines [33]

^b^PROMIS Scale v1.2 - Global Health T-score metric of 50 is the mean of US reference population [23]

^c^Food Security Survey Module: Six-Item Short Form [24] assessed via the provided protocol and categorized via the United States Department of Agriculture definitions of food security [27]

##### Primary outcomes: cardiometabolic markers

**Fig. 3:**
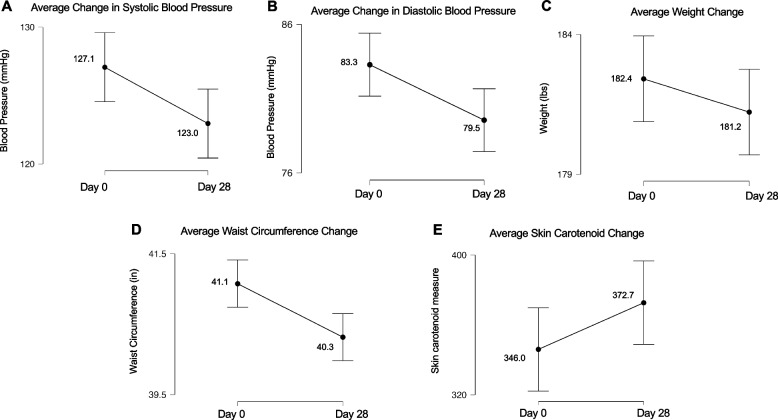
Pre-post cardiometabolic measures following a 28-day dietary approaches to stop hypertension- tailored dietary intervention

After the 28-day intervention, an improvement in systolic BP (mean difference of −4.1 ± 7.8 mmHg, p = 0.03, CI [0.55,7.69]), diastolic BP (−3.7 ± 6.4 mmHg, p = 0.01), CI [0.74, 6.73], and WC (−0.8 ± 1.1 inches, p = 0.003, CI [0.29, 1.23]) was found (Fig. 3). While the mean difference in weight (−1.2 ± 4.8 pounds, p = 0.26, CI [−0.98, 3.36]) and skin carotenoid levels (26.7 ± 74.1, p = 0.06, CI [−60.43, 7.00]) changed, results were not significant. The improvements in systolic BP, diastolic BP, and WC were consistent with a medium effect size (Cohen’s *d* = −0.53, −0.57, and −0.73, respectively) while improvements in weight and skin carotenoid status were consistent with a small effect size (Cohen’s *d* = −0.25 and 0.36, respectively).

##### Compliance to DASH eating plan guidelines

**Fig. 4:**
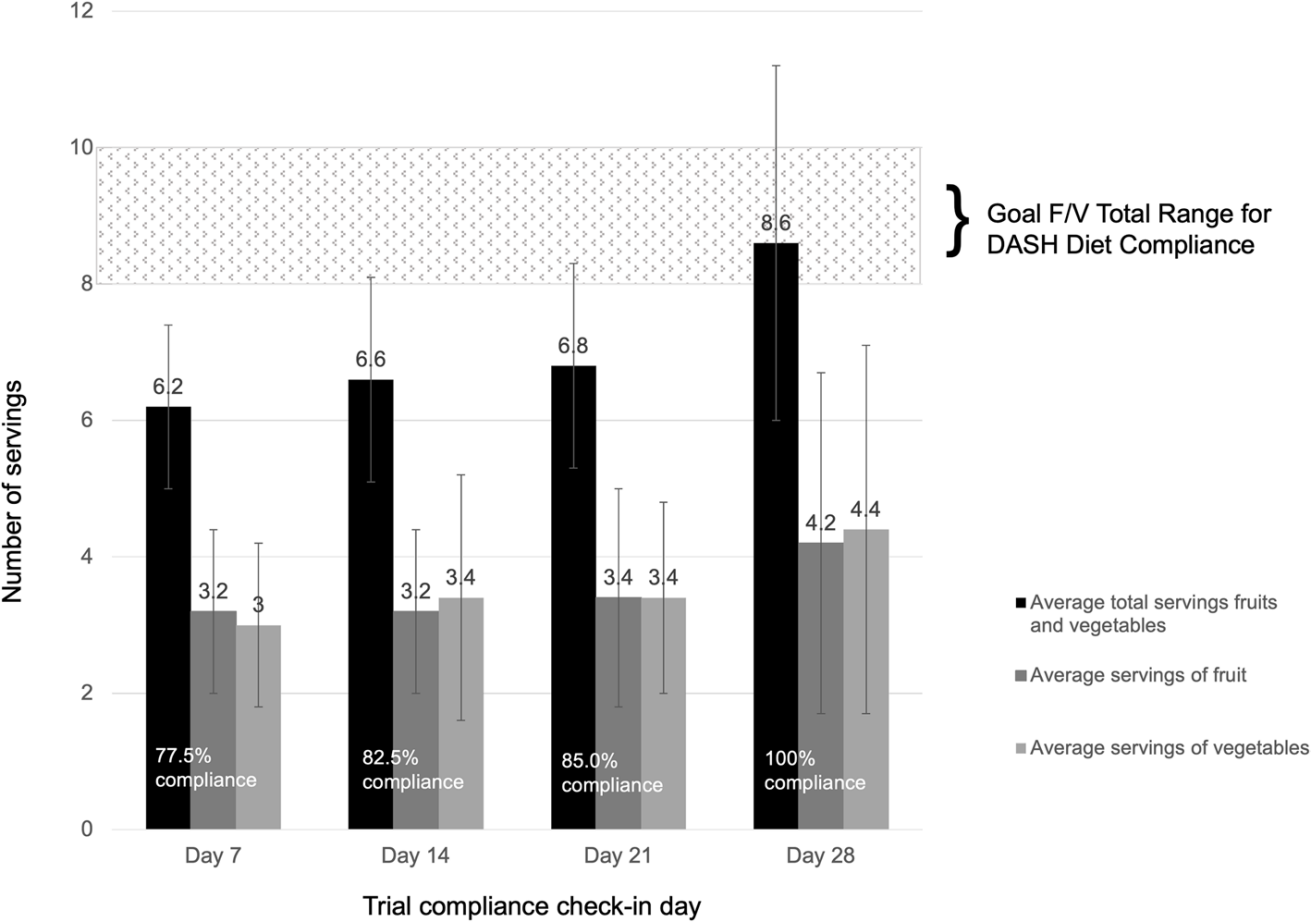
Average fruit and vegetable compliance for an open clinical trial supporting DASH eating plan recommendations

During the clinical trial, F/V intake compliance to the DASH Eating Plan F/V recommendations on average increased. On Day 0, participants reported average consumption of 5.2 servings of F/V combined prior to intervention. Average reported servings from weekly compliance checks are shown in Fig. 4. Day 7 reports from participants showed a 19.2% increase in consumption following introduction of the DASH intervention and diet education. The Day 28 compliance check showed the first marker of average reported consumption reaching F/V recommendations. Average individual compliance ranged from 4.3 ± 0.2 daily servings of F/V to 13.4 ± 0.4 daily servings of F/V combined in which 13 of the 21 participants averaged over 75% on recommended F/V intakes over the 28 days. Participants reported an equal average intake of vegetables and fruits (3.5 servings) over the course of the trial.

## Discussion

Using CBPR methods, this study involved Hispanic/*Latine* participants in the development, evaluation and implementation of a medically-tailored nutrition intervention promoting DASH Eating Plan alignment that is culturally appropriate. This iterative process led to the creation of the DASH box, which was utilized as the intervention of a single-arm pilot study. Among 21 participants completing the trial, our findings demonstrate high retention (91.3% completion rate), high average reported compliance (greater than 75%), and significant improvements in cardiometabolic markers in a 28-day free-living environment. Of importance, these preliminary findings show a clinically meaningful reduction in systolic BP (p = 0.03), diastolic BP (p = 0.01), and WC (p < 0.01) and non-significant but anticipated directional changes for weight and skin carotenoid status. The significant reductions in BP parallel findings from the multicenter Hispanic Community Health Study/Study of Latinos showing increased adherence to the DASH Eating Plan was associated with lower systolic and diastolic BP outcomes [44] and other studies working to reduce chronic disease disparities via nutrition interventions [20–23, 45].

While culturally-sensitive nutrition interventions increase effectiveness of the desired outcomes [46], Sweeny et al. noted challenges in development of culturally-appropriate meal kits, as differences in food preferences for Hispanic/*Latine* populations varied by country of origin [17]. The interviews conducted in Phase 2 supported the need for personalized interventions to capture differences in preferences between participants [18], which were implemented via a F/V needs survey. Although average F/V intake compliance in Phase 3 did not reach 100% until the final clinical trial week, supporting the participants with recipes and guidance on days 7, 14 and 21 for the use of the DASH box items may have increased compliance; research shows adherence to dietary interventions is higher when the intervention is aligned with the participant’s typical and cultural eating patterns [46–48].

Food access in urban areas is arguably higher than rural areas, however, access to basic needs such as food, is decreased for individuals with lower socioeconomic status [49]. Moreover, nutrition security, or the ability to obtain foods that prevent and treat chronic diseases [50], is lower due to increased food swamps [49]. Based on findings of our Phase 2 interviews, our study worked to reduce barriers to F/V access and use in an immigrant Hispanic/*Latine* community experiencing limitations to nutritious food due to low income, time constraints, and transportation challenges. These inequitable food environments, especially prevalent in marginalized communities [49, 51], create unequal health outcomes, especially for individuals with CVD [52]. The results in Phase 3 demonstrate that removal of the aforementioned barriers can increase nutrition security and support the significant improvement of cardiometabolic markers associated with the DASH Eating Plan for individuals with HTN. Corsino et al. tested a culturally adapted weight loss intervention for Hispanic/*Latine* adults with a recommendation to adhere to the DASH Eating Plan, finding no significant change in vegetable, low-fat dairy, and fat intakes, while fruit and grain intakes decreased [53], making our findings that barriers hinder F/V intake valuable.

The open clinical trial in Phase 3 also supported knowledge of areas for improvement based on our participants’ feedback. We identified that participants did not achieve average compliance until Day 28 of the study. Parallel to other free-living studies [54–57], our findings indicate that despite the clinical trial taking place in a free-living environment, nutrition interventions are still feasible to achieve desired clinical outcomes. However, participants requested more 1-on-1 medical nutrition therapy counseling to support the changes in a free-living environment. Other research focused on medical nutrition therapy for individuals with cardiovascular disease found lack of information regarding the dietary intervention to be one of the barriers to adhering to the treatment [58]. Future studies will likely include more targeted medical nutrition therapy for stronger support for diet adherence throughout the study.

This study demonstrates how structural racism, in this case for immigrant Hispanic/*Latine* communities, perpetuates food insecurity and therein, impacts health outcomes. Making medically-tailored meal kits culturally-appropriate and accessible promotes restorative justice and health equity, as research shows acculturation within US immigrant populations is linked to increased chronic disease risk [59–61] and decreased alignment with DASH Eating Plan recommendations [60]. Specifically, F/V intakes are necessary within the DASH Eating Plan recommendations, as F/V are rich dietary source of carotenoids; however, acculturation and a higher number of years in the US is associated with lower consumption of F/V [59, 62] and serum carotenoid concentrations [60]. Adapting medically-tailored meals kits to meet the cultural appropriateness of the marginalized population may be a way to remediate the present paradox viewing traditional Hispanic/*Latine* foods, for example, as “unhealthy” and American foods as “healthy” [18, 59] and lifestyle-related risk factors for chronic disease related to diet quality [63]. Moreover, acculturation influences the frequency of meals consumed away from home [64], but home-cooked meals are generally more nutrient dense [62] and can support the preservation of cultural cooking traditions. By providing both individual and family access to F/V in the DASH box, participants were enabled to prepare familiar foods of traditional significance for their families, potentially supporting lasting cultural and nutritional effects generationally.

A strength of this study is the novel use of CBPR methods as a phased approach to design a culturally-appropriate, medically-tailored DASH box for Hispanic/*Latine* individuals managing HTN. While the limitations impacting free-living pilot study findings are small sample sizes, single community clinic site recruitment, self-reported F/V serving adherence, and low distribution amongst different Hispanic/*Latine* ethnicities, the potential health equity impact is strong. Additionally, because this was a pilot study, sample size calculations were not conducted, which makes it difficult to detect meaningful differences or generalize findings. This project can model the use of CBPR methods to support further nutrition intervention research and resource development for marginalized communities facing food insecurity and barriers to health literacy, which lead to inequities in chronic disease incidence, morbidity, and mortality. With the recent growth in using Food as Medicine to support people with chronic disease, future research can leverage these results to develop more culturally-tailored interventions to improve participant compliance and satisfaction. Moreover, these qualitative and quantitative results can support the design of larger studies that are fully powered to provide stronger evidence for Food as Medicine.

As participants volunteered to partake in the survey, interviews, and intervention, selection bias may be present. Additionally, self-reported data, especially for F/V consumption, can introduce bias; future studies would benefit from a more objective measure of F/V consumption, such as direct measurement or photo recording. Other potential uncontrolled confounders for this free-living trial include any undocumented change in BP medication, increased physical activity post-CVD education, and/or an unclear participant understanding of portion sizes post-portion size education. These results show positive outcomes for participants; however, future studies should consider feasibility during a longer intervention period to determine plausibility of continued adherence and natural nature of variations in BP.

## Conclusions

The phased, iterative process used in this pilot study provides contributions regarding culturally-appropriate interventions for chronic disease management, suggesting a medically-tailored DASH box with consideration and personalization to patient needs may be effective in improving BP and other cardiometabolic risk factors for Hispanic/*Latine* individuals with HTN. An emphasis should be placed on identifying facilitators and barriers to eating pattern alignment to support optimal adherence. As noted, when reducing the barriers to food access, such as transportation, cost (via providing additional weekly servings of F/V and staple items), nutrition education, and time spent grocery shopping, alignment with the DASH Eating Plan increases; adding a cultural and personal tailoring is a potential facilitator to consider for tailored meals or groceries. While average compliance to meet the DASH Eating Plan was not achieved until Day 28 of the study, cardiometabolic health markers were improved. Therefore, addressing societal inequities that exist on an individual, interpersonal, and community level from reduced access to F/V recommended for HTN can support a reduction in health disparities present for those managing chronic diseases. There is a continued need to provide health equity in nutrition interventions as diet is a modifiable lifestyle-related risk factor for many chronic diseases such as HTN.

## Supplementary Information

Supplementary Material 1.

## Data Availability

The datasets generated and/or analyzed during the current study are not publicly available due to sensitive information regarding the documentation status of immigrants, but are available from the corresponding author on reasonable request.

## Abbreviations

BP: Blood pressure
CBPR: Community-based participatory research
CVD: Cardiovascular disease
DASH: Dietary Approaches to Stop Hypertension
F/V: Fruits and vegetables
HTN: Hypertension
mmHg: Millimeters of mercury
REDCap: Research Electronic Data Capture
SD: Standard deviation
SE: Standard error
US: United States
USDA: United States Department of Agriculture
WC: Waist circumference
