# Supplementary material for: Developing and evaluating a culturally-appropriate food kit for increased access to fruits and vegetables and DASH eating plan alignment in immigrant Hispanic/Latine individuals with hypertension: a pilot study
Source: BMC Nutr. 2025 May 16;11:97. doi: 10.1186/s40795-025-01089-z (PMC12082931; doi:10.1186/s40795-025-01089-z)
Supplement: Supplementary file 1 — Supplementary Material 1. [file 40795_2025_1089_MOESM1_ESM.docx]

**Phase 1 Survey**

1. **Age (how old are you)**[26]**:** (open)
2. **Are you of Hispanic, Latino, or Spanish origin?** [26] No, not of Hispanic, Latino, or Spanish origin; Yes, Mexican, Mexican Am., Chicano; Yes, Puerto Rican; Yes, Cuban; Yes, another Hispanic, Latino, or Spanish origin - for example, Salvadoran, Dominican, Colombian, Guatemalan, Spaniard, Ecuadorian, etc.
3. **Race (select all that apply)**[26]**:** White - for example, German, Irish, print origins. English, Italian, Lebanese, Egyptian, etc.; Black or African Am. - for example, African American, Jamaican, Haitian, Nigerian, Ethiopian, Somali, etc.; American Indian or Alaska Native - for example, Navajo Nation, Blackfeet Tribe, Mayan, Aztec, Native Village of Barrow Inupiat Traditional Government, Nome Eskimo Community, etc.; Chinese; Filipino; Asian Indian; Vietnamese; Korean; Japanese; Other Asian - for example, Pakistani, Cambodian, Hmong, etc.; Native Hawaiian; Samoan; Chamorro; Other Pacific Islander - for example, Tongan, Fijian, Marshallese, etc.; Some other race: please specify_____________________
4. **Biological Sex assigned at birth** [26]**:** Female; Male; Intersex; None of these describe; Prefer not to answer; Other ___________________
5. **What terms best express how you describe your gender identity** [26]**:** Man; Woman; Non-binary; Transgender; Genderqueer; Genderfluid; Gender Variant; Questioning or unsure of your gender identity; None of these describe me; Prefer not to answer
6. **Sexual Identity** [26]**:**  Gay; Lesbian; Straight; Bisexual; Queer; Polysexual/omnisexual/sepiosexual/pansexual; Asexual, Two-spirit; have not figured out/in the process of figuring out my sexuality; Do not think of yourself as having sexuality; Do not use labels to identify yourself; None of these describe me; Prefer not to answer
7. **Birthplace** [26]**:** In the United States: Print Name of State_________________; Outside the United States - Print U.S. Territory (e.g., Puerto Rico, U.S. Virgin Islands, Guam) or name of foreign country______________________
8. **Current City/State/Zip Code** [26]**:**  _______________________________
9. **What is the highest grade or level of school you have completed or the highest degree you have received?** [26] (open)
10. **We would like to know about what you do --are you working now, looking for work, retired, keeping house, a student, or what?**[26] Working now; only temporarily laid off, on sick leave or maternity leave; Looking for work/unemployed; Retired; Disabled: permanently or temporarily; Keeping house; Student; Other (SPECIFY):
11. **Since you speak a language other than English at home, we are interested in your own opinion of how well you speak English. Would you say you speak English...? [26]** Very well; Well; Not well; Not at all; Refused; Don't know
12. **Please tell me whether the statement was OFTEN, SOMETIMES, or NEVER true for (you/you and the other members of your household) in the last 12 months** [28]**:**

- **The food that (I/we) bought just didn't last, and (I/we) didn't have money to get more. Was that often, sometimes, or never true for (you/your household) in the last 12 months?** Often true; Sometimes true; Never true; Don't know; Refused
- **(I/we) couldn't afford to eat balanced meals. Was that often, sometimes, or never true for (you/your household) in the last 12 months?** Often true; Sometimes true; Never true; Don't know; Refused
- **In the last 12 months, since (date 12 months ago) did (you/you or other adults in your household) ever cut the size of your meals or skip meals because there wasn't enough money for food?** Yes; No; Don't know; Refused
- **If yes, how often did this happen—almost every month, some months but not every month, or in only 1 or 2 months?** Almost every month; some months but not every month; In only 1 or 2 months; Don't know; Refused
- **In the last 12 months, did you ever eat less than you felt you should because there wasn't enough money to buy food?** Yes; No; Don't know; Refused
- **In the last 12 months, were you ever hungry but didn't eat because you couldn't afford enough food?** Yes; No; Don't know; Refused

1. **Have you ever been treated for the following conditions (select all that apply)**[27]**:** Hypertension, High cholesterol, pre-diabetes, diabetes, heart attack, stroke, deep vein thrombosis, diabetic retinopathy
2. **In general,**

- **Would you rate your health as** [27]**:** Excellent; Very good; Good; Fair; Poor
- **Would you say your quality of life is** [27]**:** Excellent; Very good; Good; Fair; Poor
- **How would you rate your physical health?**[27]**:** Excellent; Very good; Good; Fair; Poor
- **How would you rate your mental health, including your mood and your ability to think?**[27]**:** Excellent; Very good; Good; Fair; Poor
- **How would you rate your satisfaction with your social activities and relationships?**[27]**:** Excellent; Very good; Good; Fair; Poor
- **Please rate how well you carry out your usual social activities and roles. (This includes activities at home, at work and in your community, and responsibilities as a parent, child, spouse, employee, friend, etc.)**[27]**:** Excellent; Very good; Good; Fair; Poor

1. **To what extent are you able to carry out your everyday physical activities such as walking, climbing stairs, carrying groceries, or moving a chair?** [27]**:** Completely; Mostly; Moderately; A little; Not at all
2. **In the past 7 days…**

- **How often have you been bothered by emotional problems such as feeling anxious, depressed or irritable?**[27]**:** Never; Rarely; Sometimes; Often; Always
- **How would you rate your fatigue on average?** [27]**:** Never; Rarely; Sometimes; Often; Always
- **How would you rate your pain on average? (0= no pain, 10= Worst pain imaginable** [27]**:** (open)

1. **Food allergies (please list):** (open)
2. **Dietary Preferences:** vegan, vegetarian, pescatarian (eats mainly plant proteins and fish), ovo-vegetarian (eats mainly plant proteins and eggs), lacto-ovo vegetarian (eats mainly plant proteins and eggs/dairy), flexitarian (eats mainly plant proteins, but will occasionally eat meat), no restrictions

**From the list below, please place an “x” by the appropriate boxes of fruits you would consume if available:**

| - Apples - Avocado - Bananas - Blackberries - Blueberries - Cape gooseberries/Ground cherries - Cherries - Coconut - Dragonfruit - Grapes - Guava - Mangoes - Melon (honeydew, cantaloupe) - Mulberries - Lemon - Lime | - Oranges/tangerine/clementine - Papaya - Pears - Peaches - Pepino dulce - Pineapple - Plums - Pomegranate - Prickly pear cactus fruit - Raspberries - Sapote - Starfruit - Strawberries - Watermelon - Other _________________ - Other _________________ - Other _________________ |
| --- | --- |

Please list your top 5 preferred fruits to eat by themselves or to mix in with other dishes:

1. ___________________
2. ___________________
3. ___________________
4. ___________________
5. ___________________

**From the list below, please place an “x” by the appropriate boxes of vegetables you would consume if available:**

| - Artichoke - Asparagus - Beets - Beans (black eyed pea, pinto, other: ________________) - Broccoli - Cabbage (red, green) - Cactus (nopales, other: _____________) - Carrot - Cauliflower - Celery - Cilantro - Collard greens - Corn - Cucumber - Eggplant - Green or yellow beans - Hearts of palm - Hominy - Jicama - Kale - Lettuce (butter leaf, romaine) - Mushrooms | - Okra - Olives - Onions - Peas - Peppers (Bell- red, green, orange, yellow; poblano) - Peppers (Hot- jalapeno, habanero, chili, other: ____________) - Potatoes (purple, white, yellow) - Pumpkin - Spinach - Squash (butternut, acorn, chayote, other: _______________) - Sweet potatoes/Yam - Taro - Tomato - Tomatillo - Yucca - Zucchini (yellow or green) - Other _________________ - Other _________________ - Other _________________ |
| --- | --- |

Please list your top 5 preferred vegetables to eat by themselves or to mix in with other dishes:

1. ___________________
2. ___________________
3. ___________________
4. ___________________
5. ___________________
